# Changes in the Lung Microbiome following Lung Transplantation Include the Emergence of Two Distinct Pseudomonas Species with Distinct Clinical Associations

**DOI:** 10.1371/journal.pone.0097214

**Published:** 2014-05-15

**Authors:** Robert P. Dickson, John R. Erb-Downward, Christine M. Freeman, Natalie Walker, Brittan S. Scales, James M. Beck, Fernando J. Martinez, Jeffrey L. Curtis, Vibha N. Lama, Gary B. Huffnagle

**Affiliations:** 1 Division of Pulmonary and Critical Care Medicine, Department of Internal Medicine, University of Michigan Medical School, Ann Arbor, Michigan, United States of America; 2 Research Service, Department of Veterans Affairs Health Care System, Ann Arbor, Michigan, United States of America; 3 Department of Microbiology and Immunology, University of Michigan Medical School, Ann Arbor, Michigan, United States of America; 4 Department of Medicine, University of Colorado Denver, Aurora, Colorado and Medicine Service, Veterans Affairs Eastern Colorado Health Care System, Denver, Colorado, United States of America; 5 Pulmonary & Critical Care Medicine Section, Medical Service, VA Ann Arbor Healthcare System, Ann Arbor, Michigan, United States of America; The Ohio State University, United States of America

## Abstract

**Background:**

Multiple independent culture-based studies have identified the presence of *Pseudomonas aeruginosa* in respiratory samples as a positive risk factor for bronchiolitis obliterans syndrome (BOS). Yet, culture-independent microbiological techniques have identified a negative association between *Pseudomonas* species and BOS. Our objective was to investigate whether there may be a unifying explanation for these apparently dichotomous results.

**Methods:**

We performed bronchoscopies with bronchoalveolar lavage (BAL) on lung transplant recipients (46 procedures in 33 patients) and 26 non-transplant control subjects. We analyzed bacterial communities in the BAL fluid using qPCR and pyrosequencing of 16S rRNA gene amplicons and compared the culture-independent data with the clinical metadata and culture results from these subjects.

**Findings:**

Route of bronchoscopy (via nose or via mouth) was not associated with changes in BAL microbiota (p = 0.90). Among the subjects with positive *Pseudomonas* bacterial culture, *P. aeruginosa* was also identified by culture-independent methods. In contrast, a distinct *Pseudomonas* species, *P. fluorescens*, was often identified in asymptomatic transplant subjects by pyrosequencing but not detected via standard bacterial culture. The subject populations harboring these two distinct pseudomonads differed significantly with respect to associated symptoms, BAL neutrophilia, bacterial DNA burden and microbial diversity. Despite notable differences in culturability, a global database search of UM Hospital Clinical Microbiology Laboratory records indicated that *P. fluorescens* is commonly isolated from respiratory specimens.

**Interpretation:**

We have reported for the first time that two prominent and distinct *Pseudomonas* species (*P. fluorescens* and *P. aeruginosa*) exist within the post-transplant lung microbiome, each with unique genomic and microbiologic features and widely divergent clinical associations, including presence during acute infection.

## Introduction

In recent years, novel culture-independent techniques of microbial identification have permitted analysis of entire bacterial communities within the airways of patients with various diseases [1]–[4]. Lung transplantation is the only therapeutic option for many end-stage lung diseases [5]. Microbial infection and colonization have been associated with increased morbidity and mortality among lung transplant recipients, due to pneumonia or bronchiolitis obliterans syndrome (BOS) [5]–[8]. Lung transplantation and the immunosuppressive therapies it requires result in numerous changes to host defenses that may alter the microbiota of the respiratory tract [9], [10]. In a recent study of lung transplant recipients using culture-independent techniques, Willner et. al observed a negative association between the abundance of *Pseudomonas* species (spp). and the diagnosis of BOS [11], a surprising result given the numerous independent studies demonstrating that the detection of *P. aeruginosa* in respiratory cultures is a positive risk factor for the subsequent development of BOS [6], [12], [13]. Additionally, studies of the post-transplant lung microbiome have conflicted regarding the impact of transplantation on microbial diversity, with one report finding increased diversity compared to controls [14] and another reporting decreased diversity [15]. The source of these conflicting findings, as well as the clinical significance and associated clinical factors of post-transplant microbial diversity, remain undetermined.

In this study, we aimed to address these conflicting findings via culture-independent identification of microbial communities in BAL samples obtained from lung transplant recipients, stratified by clinical parameters, and non-transplant control subjects. We hypothesized that the post-transplant lung microbiome would be distinct from that of non-transplant controls, and, consistent with the dichotomous reports, would contain more than one prominent species of *Pseudomonas* that would correlate with transplant health. We also hypothesized that the diversity of post-transplant lung microbiota would not be uniform among transplant recipients and would correlate with clinically significant parameters.

## Methods

### Ethics Statement

All clinical investigations were conducted according to the principles expressed in the Declaration of Helsinki. The study protocol was approved by the institutional review boards of the University of Michigan Healthcare System and the Ann Arbor Veterans Affairs Healthcare System. All patients provided written informed consent. The institutional review boards have examined the protocols and certified that “The risks are reasonable in relation to benefits to subjects and the knowledge to be gained. The risks of the study have been minimized to the extent possible.”

### Subject enrollment

#### Lung transplant recipients

BAL samples were obtained from lung transplant recipients undergoing bronchoscopy at the University of Michigan. All lung transplant recipients at the University of Michigan were eligible for enrollment in the study. Specimens were collected consecutively between 11/1/2011 and 8/1/2012.

#### Non-transplant control subjects

Specimens were obtained from volunteers enrolled in the Lung HIV Microbiome Project who underwent research bronchoscopy at the VA Ann Arbor Healthcare System [16], [17]. All subjects were HIV-negative.

### Clinical data

Clinical data regarding lung transplant recipients was abstracted from the electronic medical record of the University of Michigan and from the Organ Transplant Information System (OTIS). BOS was defined by physiologic testing according to the International Society of Heart and Lung Transplantation guidelines [18].

### Sample acquisition and processing

Patients received conscious sedation and nebulized lidocaine. The bronchoscope was advanced via the mouth or nose and through the vocal cords. After a brief airway exam, the bronchoscope was wedged in the right middle lobe or lingula of the allograft (for surveillance bronchoscopies) or, in the case of symptomatic patients with available imaging, in the segment with the most evidence of radiographic abnormality. In non-transplant control subjects, the bronchoscope was wedged in the right middle lobe and lingula. BAL was performed with instillation of between 120 and 300 ml of sterile isotonic saline. Samples were stored on ice, centrifuged at 13,000 RPM for 30 minutes (Hermle Z 231 M microcentrifuge) in dolphin-nosed Eppendorf tubes and stored at −80**°**C until the time of DNA extraction. All samples obtained from transplant subjects were processed by the University of Michigan Microbiology Laboratory for routine microbial analysis (bacterial, fungal and AFB culture). For bacterial culture, BAL fluid was plated on chocolate, sheep blood and MacConkey agar plates and incubated for ≥72 hours. Bacteria were identified and reported if they grew more than 10^4^ colony forming units (CFU) per mL, or if under 10^4^ CFU/mL but were a single gram negative bacillus species and the only reportable pathogen.

### DNA isolation

Genomic DNA was extracted from BAL pellets resuspended in 360 µl ATL buffer (Qiagen DNeasy Blood & Tissue kit) and homogenized in UltraClean fecal DNA bead tubes (MO-BIO, Carlsbad, CA) using a modified protocol previously demonstrated to isolate bacterial DNA [19].

### Quantitative Polymerase Chain Reaction (qPCR)

Quantification of bacterial 16S rDNA was performed by real-time PCR utilizing TaqMan hydrolysis probes on a Roche 480 LightCycler. Degenerate bacterial 16S rDNA specific primers were targeted to the V1-V2 regions of the 16S rDNA gene using the following sequences: 5′-AGAGTTTGATCCTGGCTCAG-3′ (forward); 5′-CTGCTGCCTYCCGTA-3′ (reverse); (5′-FAM-TA+ACA+CATG+CA+AGTC+GA- BHQ1-3′ (probe). [20]–[22]. The probe was developed for the primer BSR65/17 landing site using the following sequence: 5′-TCGACTTGCATGTRTTA-3′. 16S clones derived from a *Haemophilus* species were used for generation of a standard curve. After an initial denaturation of five minutes at 95°C, 40 cycles of amplification were performed: 30 seconds at 94°C, 30 seconds at 50°C and 30 seconds at 72°C. A final elongation step was performed at 72°C.

### 454 Pyrosequencing

The V3–V5 hypervariable regions of the bacterial 16S rRNA gene were sequenced in the V5–V3 direction using barcoded primer sets corresponding to 357F and 926R [23]. These barcoded primers were originally developed by the Broad Institute. Primary PCR cycling conditions were 95°C for two minutes, followed by 20 cycles of touchdown PCR (95°C 20 seconds, followed by an annealing for 30 seconds beginning at 60°C and decreasing one degree every two cycles until 50°C, and an elongation of 72°C 45 seconds), then 20 cycles of standard PCR (95°C for 20 seconds, 50°C for 30 seconds, and 72°C for 45 seconds), and finished with 72°C for 5 minutes. Quality control and sequencing was carried out at the University of Michigan, using the Roche 454 GS Junior according to established protocols [24]. Pre-procedure bronchoscope rinse controls, reagent water controls and mock community standards were analyzed with each sequencing run as quality controls.

### Data analysis

Sequence data were processed and analyzed using the software mothur v.1.27.0 according to the Standard Operating Procedure for 454 sequence data (http://www.mothur.org) using a minimum sequence length of 250 basepairs [25]. A shared community file and a phylotyped (genus-level grouping) file were generated using operational taxonomic units (OTUs) binned at 97% identity generated using the dist.seqs, cluster, make.shared and classify.otu commands in mothur. No subsampling was performed and all subsequent phylogenetic analysis was performed in R. Amounts of bacterial DNA detected in reagent water controls were small relative to BAL and mock community specimens (discussed in results below). OTUs detected in reagent water controls were removed from all BAL specimens prior to analysis. OTU numbers were arbitrarily assigned in the binning process and are referred to throughout the manuscript in association with their most specified level of taxonomy.

These files, along with the files containing the taxonomic information for the OTUs, were imported and further analyzed in R using the R-package vegan 2.0–4 for diversity analyses and ordinations, and a custom R script for sorting classification results into tables. Classification of OTUs was carried out using the mothur implementation of the Ribosomal Database Project (RDP) Classifier and the RDP taxonomy training set 9 (fasta reference  =  trainset9_032012.pds.fasta, taxonomy reference  =  trainset9_032012.pds.tax), available on the mothur website. A mean of 1476±703 high-quality reads were obtained per BAL specimen. 1109 unique OTUs were identified across all the specimens. For relative abundance and ordination analysis, samples were normalized to the percent of total reads and we restricted analysis to OTUs that were present at greater than 1% of the sample population; all OTUs were included in diversity analysis. Sequences are available online at the NIH Sequence Read Archive (http://www.ncbi.nlm.nih.gov/sra, accession numbers 2419687–2419764).

### Microbe-Specific PCR

Select BAL specimens were analyzed via PCR using microbe-specific primers. *Pseudomonas aeruginosa*-targeted primers used were PA-SS-F (5′-GGG GGA TCT TCG GAC CTC A-3′, location: 189-206) and PA-SS-R (5′-TCC TTA GAG TGC CCA CCC G-3′, location: 1124-1144) [26]. These primers were targeted at species-specific signature sequences in the 16S rDNA variable regions 2 and 8 and validated in our laboratory against numerous *Pseudomonas* species. For these primers, the initial denaturization step was at 95 C for 10 minutes, followed by 25 cycles of 94 C for 20 seconds, 58 C for 20 seconds and 72 C for 40 seconds with a final extension step of one minute at at 72 C. *Pseudomonas fluorescens*-targeted primers used were 16SPSEfluF (5′-TGC ATT CAA AAC TGA CTG-3′, location: 493-510) and 16SPSER (5′-AAT CAC ACC GTG GTA ACC G-3′, location: 1323–1338) [27]. For these primers, the initial denaturization step was at 95 C for 10 minutes, followed by 5 cycles of 94 C for 45 seconds, 55 C for 1 minute and 72 C for 2 minutes. This was followed by 35 cycles of 92 C for 45 seconds, 60 C for 45 seconds, 72 C for 2 minutes and a final extension step of 72 C for 2 minutes. Final cooling was performed at 4 C.

### Phylogenetic Tree Generation

Bacterial genomes were uploaded into DNASTAR SeqBuilder (Lasergene). Sequences corresponding to the V3–V5 region of the 16S rRNA gene were aligned to each genome. A fasta-format document containing V3–V5 sequences was uploaded into MAFFT v.7, an online multiple sequence alignment program (http://mafft.cbrc.jp/alignment/server/) [28]. Details regarding the tree-building algorithm can be found at http://mafft.cbrc.jp/alignment/software/algorithms/algorithms.html. After sequence alignment, bootstrapping was performed and 1000 re-sample iterations were executed before generation of the final phylogenetic tree.

### Statistical analysis

Statistical analyses were performed using Prism 5 (GraphPad Software) for ANOVA, t-test and regression analysis, and vegan and R for all diversity, rank abundance and ordination analyses. ANOVA-like permutation testing of constrained ordinations, both redundancy analysis (RDA) and by canonical correspondence analysis (CCA), was performed using the anova.cca function in the R package vegan. Significant differences in community membership identified via constrained ordination were confirmed using PERMANOVA (permutational multivariate analysis of variance) via the *adonis* function in vegan.

### Study population

46 bronchoscopies were performed on 33 lung transplant recipients (**Table 1**). Six patients underwent two bronchoscopies, two underwent three and one patient underwent four; the remaining 24 patients underwent one bronchoscopy. Of the 46 specimens, two had minimal 16S bacterial DNA signal and were excluded from subsequent diversity, ordination, and rank-abundance analysis, but were included in relative 16S rDNA qPCR comparisons.

**Table 1:** Patient and Bronchoscopy Characteristics.

Patient characteristics					
		Total (33)	Asymptomatic (17)	Symptomatic (16)	p
**Patient demographics:**	Male	29 (79%)	16 (94%)	10 (62%)	*0*.*04*
	Age	20–66 (49.9±15.8)	22–66 (52.6±17.2)	20–62 (47.0±14.4)	0.32
	Age at transplant	19–65 (48.7±15.5)	22–65 (51.6±16.6)	20–61 (45.7±14.2)	0.29
	Days post-transplant	26–2626 (431.7±575)	26–2626 (407.4±661.8)	58–1827 (457.6±487.5)	0.81
**Type of transplant:**	Bilateral lung	26 (79%)	11 (65%)	15 (94%)	0.08
	Single lung	13 (21%)	6 (35%)	1 (6%)	0.08
**Pre-transplant diagnosis:**	Pulmonary fibrosis	13 (39%)	6 (35%)	7 (44%)	0.73
	Cystic fibrosis	8 (24%)	4 (24%)	4 (25%)	1
	COPD	5 (15%)	4 (24%)	2 (12%)	0.65
	Other	7 (21%)	3 (18%)	3 (19%)	1
**BOS:**	BOS (ever)	7 (21%)	1 (6%)	6 (38%)	*0.04*
	BOS (at time of bronchoscopy)	4 (12%)	0 (0%)	4 (25%)	*0.04*
**Bronchoscopy characteristics**					
		**Total (44)**	**Asymptomatic (23)**	**Symptomatic (21)**	**p**
**Antibiotics:**	Antibiotics within one month	28 (64%)	12 (52%)	16 (76%)	0.13
	Antibiotics within one week	19 (43%)	8 (35%)	11 (52%)	0.36
	Antibiotics at bronchoscopy	16 (36%)	6 (26%)	10 (48%)	0.21
**Pathology:**	Acute cellular rejection (A-grade)	5 (11%)	2 (9%)	3 (14%)	0.66
	Acute airway inflammation (B-grade)	2 (4%)	2 (9%)	0 (0%)	0.49
	Organizing pneumonia	6 (14%)	3 (13%)	3 (14%)	1

Depending on variable type, data are presented as either *N (% of group)* or as *range (mean ± standard deviation)*.

26 bronchoscopies were performed on 26 non-transplant control subjects, all lacking known history of lung disease (**Table 2**). All 26 specimens were included in all analyses.

**Table 2:** Non-transplant Control Subject Characteristics (n = 26).

Male	8 (31%)
Age	18–75 (40.2±16.6)
Current Smoker	5 (19%)
Former Smoker	4 (15%)
FEV1% predicted	59–150 (103.2±16.9)

For the purposes of analysis, transplant recipients were considered asymptomatic if the bronchoscopy was performed as a scheduled surveillance bronchoscopy (performed routinely at the University of Michigan following transplantation at six weeks, three months, six months and 12 months) and the patient had no acute complaints of cough, dyspnea, fever or increased sputum production and was not undergoing bronchoscopy for a newly appreciated radiographic infiltrate or decrease in lung function.

### Patient Demographics

Most transplant recipients were male and had undergone bilateral lung transplantation (**1**). The most common pre-transplant diagnosis was pulmonary fibrosis, followed by cystic fibrosis (CF) and chronic obstructive pulmonary disease (COPD). Most bronchoscopies (67%) were performed within one year of transplantation.

When compared to patients who were asymptomatic at the time of bronchoscopy, symptomatic patients were more likely to be female and to have a history of BOS (p<0.05, **Table 1**). Symptomatic and asymptomatic patients did not differ significantly with regard to time since transplant or pre-transplant diagnosis (p>0.05, **Table 1**). A higher fraction of symptomatic subjects had recent antibiotic exposure and positive BAL bacterial culture (both *Pseudomonas* and otherwise), though these did not meet statistical significance (p>0.05, **Table 1**, **Table 3**). The most commonly prescribed classes of antibiotics were fluoroquinolones, tetracyclines and macrolides. Only three subjects received nebulized tobramycin in the time prior to BAL. There were no significant differences in immunosuppression between the symptomatic and asymptomatic subjects.

**Table 3:** Culture Results of Lung Transplant BAL Specimens.

	Total (44)	Asymptomatic (23)	Symptomatic (21)	p
Positive bacterial growth (any)	35 (80%)	19 (83%)	16 (76%)	0.72
Positive bacterial growth (>10K CFU/mL)	18 (41%)	7 (30%)	11 (52%)	0.22
Positive *Pseudomonas aeruginosa* growth	9 (20%)	2 (9%)	7 (33%)	0.06

## Results

### BAL from Lung Transplant Recipients Contains Distinct Bacterial Microbiota from that of Non-transplant Control Subjects

**Figure 1:**
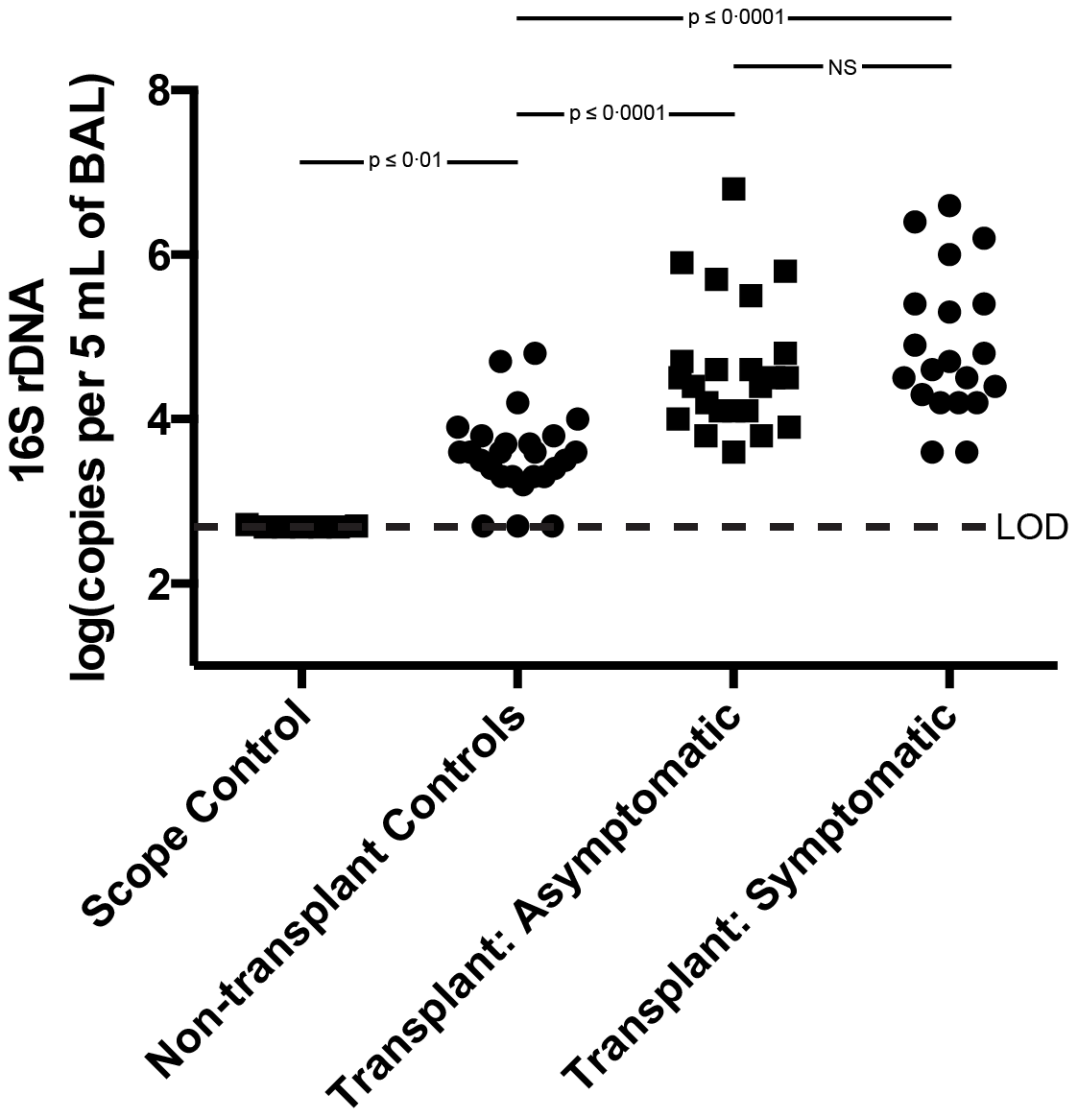
16S rRNA gene qPCR of DNA prepared from unfractionated BAL samples. The number of copies of bacterial 16S rRNA genes per 5's multiple comparisons test.

Bacteria levels in the samples from the various groups were determined by quantifying 16S rRNA gene copy numbers in 5 ml of unfractionated BAL. The amount of bacterial DNA detected in the pre-procedure bronchoscope rinse was at or near the limit of detection (**Figure 1**). In healthy, non-smoking individuals (non-transplant controls), the amount of bacteria in the BAL was significantly higher than in the pre-procedure control samples. Most importantly, the bacteria levels in the BAL specimens from lung transplant recipients were significantly higher than those in the healthy controls **(** **Figure 1** **).** On average, there was 15-fold more bacterial in the BAL specimens from transplant recipients than in those from non-transplant controls, with some samples 1000 fold higher **(** **Figure 1** **)**. There was no significant difference in bacterial 16S rRNA gene levels between symptomatic and asymptomatic transplant recipients. Thus, the BAL from lung transplant recipients contained significantly more bacteria than that from non-transplant control subjects.

**Figure 2:**
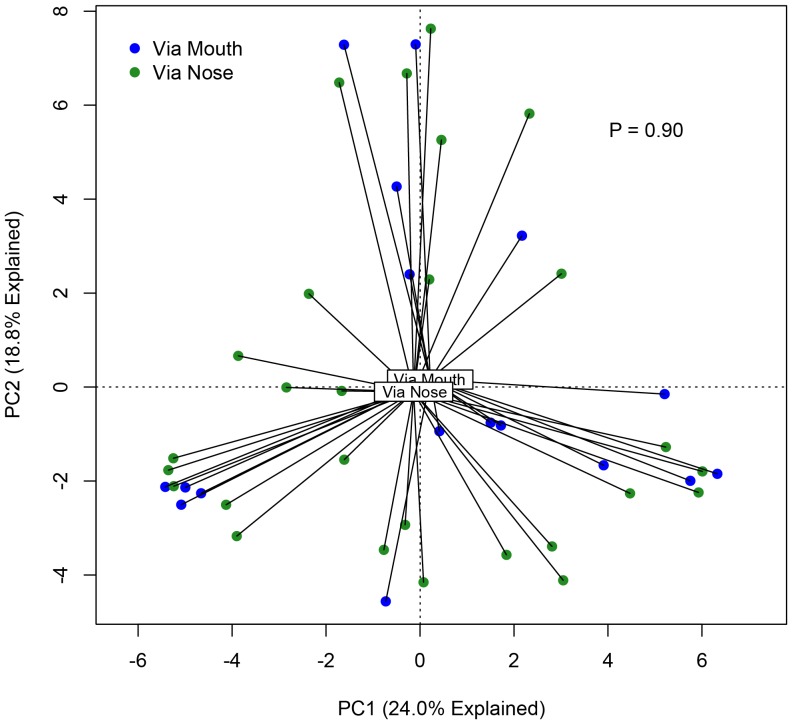
Comparison of transplant BAL specimens obtained via nasal and oral routes of bronchoscopy. Unsupervised principal component analysis (PCA) labeled by route of bronchoscope insertion. Group dissimilarity tested using ANOVA permutation test of ordination constrained by route of insertion.

Because bronchoscopies of transplant subjects were performed both via the nose and mouth, which contain distinct microbiota, and given the possibility of upper respiratory tract contamination of BAL specimens, we asked whether route of bronchoscope insertion was associated with differences in BAL microbiota. The majority (61%) of bronchoscopies performed on transplant subjects were performed via nasal insertion of the bronchoscope; the remainder were performed via an oral route. We employed the data visualization technique of Principal Components Analysis (PCA) to compare the bacterial communities in these specimen groups. We detected no spatial separation of BAL specimens obtained via the nose and those obtained via the mouth (**Figure 2** **).** The two specimen groups were not statistically distinct when tested either via ANOVA-like permutation testing of constrained ordination (both redundancy analysis [RDA] and canonical correspondence analysis [CCA]) or via the *adonis* (PERMANOVA) function in vegan (p = 0.90). Thus route of bronchoscope insertion had no appreciable effect on BAL microbiota, arguing against significant contamination via upper respiratory tract microbiota.

**Figure 3:**
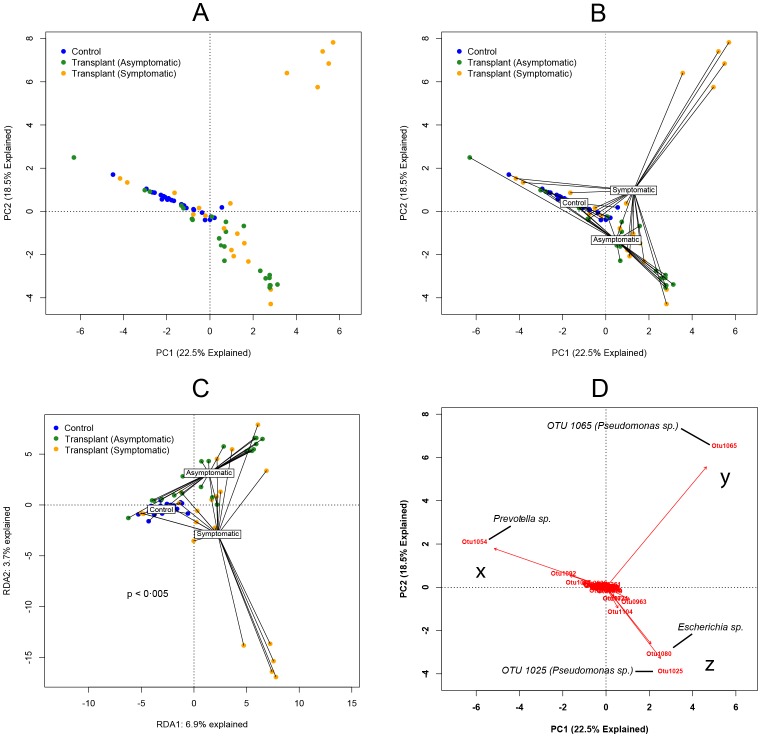
Ordination analyses of bacterial communities detected in BAL samples. Unsupervised principal component analysis (PCA): labeled by specimen group (A) and with specimen group centroids labeled (B) C: Ordination constrained by specimen group (RDA). Group dissimilarity tested using ANOVA permutation test. D: Biplot analysis of PCA plot with prominent OTUs labeled.

Our next objective was to compare the composition of the BAL microbiota between transplant subjects and non-transplant controls, as well as between symptomatic and asymptomatic recipients. Using an unconstrained PCA of all samples (**Figure 3A**), we observed spatial separation of the three pre-specified subject groups (non-transplant controls, asymptomatic transplant recipients and symptomatic transplant recipients), though location of each group's members within the ordination was heterogenous. This separation of specimens by subject group was apparent when each group's centroid was plotted (**Figure 3B**), implying differences in the collective microbiota of each group when compared to the others. To test for statistical significance of these findings, we performed ANOVA-like permutation testing of constrained ordination, both by RDA and CCA. RDA demonstrated that significantly different microbial communities were associated with each of the subject groups (control, symptomatic recipients or asymptomatic recipients) (p<0.005, **Figure 3C**). This significance persisted when controlled for pretransplant diagnosis, time since transplant and FEV1 at the time of bronschoscopy (p<0.005 for all), and was confirmed using *adonis* (PERMANOVA) function in vegan (p<0.005) and corresponding multivariable analyses. When constrained by clinical parameters, only the presence of BOS at the time of bronchoscopy (p = 0.01) or of BOS at any point (p = 0.02) were associated with significant differences in BAL microbiota composition by ordination (**Table 4**). The significance of the distinct microbiota detected in subjects with BOS persisted when tested using adonis (p = 0.03) and when controlled for FEV1 at the time of bronchoscopy (p = 0.02) but not when controlled for time since transplant (p = 0.06). No significant differences were found when ordination was constrained by any other clinical parameter (p>0.05 for all) (**Table 4**). These analyses demonstrated that significant differences in the BAL bacterial communities could be identified between non-transplant, asymptomatic and symptomatic subjects, with BOS associated with significant differences in BAL microbiota among lung transplant recipients.

**Table 4:** Significance of Comparisons of Lung Transplant Recipients by Diversity Analysis and Constrained Ordination.

Clinical Characteristic		Constrained Ordination Significance (*p*)	Shannon Diversity Index Significance (*p*)
**Transplant/ Patient Factors**	Pre-transplant diagnosis	>0.05	>0.05
	Type of transplant (single/bilateral)	>0.05	>0.05
	Sex	>0.05	>0.05
	Age	>0.05	>0.05
	Time over one year	>0.05	>0.05
	Antibiotics within 30 days	>0.05	>0.05
	Antibiotics within 7 days	>0.05	>0.05
	Antibiotics at time of BAL	>0.05	>0.05
	Pneumocystis prophylaxis agent	>0.05	>0.05
**BAL Results**	Bacterial DNA (16S qPCR)	>0.05	**p≤0.001**
	BAL neutrophilia	>0.05	**p = 0.017**
	Bacterial growth (>10^4^ CFU/mL)	>0.05	**p≤0.001**
	Bacterial growth (any)	>0.05	>0.05
	Fungal growth	>0.05	>0.05
	Acute cellular rejection	>0.05	>0.05
**Airway Factors**	FEV1 under 70% predicted	>0.05	>0.05
	BOS (ever)	0.0225	>0.05
	BOS (at bronchoscopy)	0.01	>0.05

Statistical significance of comparisons of Shannon Diversity Indices was determined using Student's t-test and ANOVA for categorical variables and linear regression analysis for continuous variables. Constrained ordination was performed using Canonical Correspondance Analysis, and significance was determined using ANOVA-like permutation testing.

### Decreased Bacterial Diversity is Associated with Evidence of Acute Bacterial Infection

**Figure 4:**
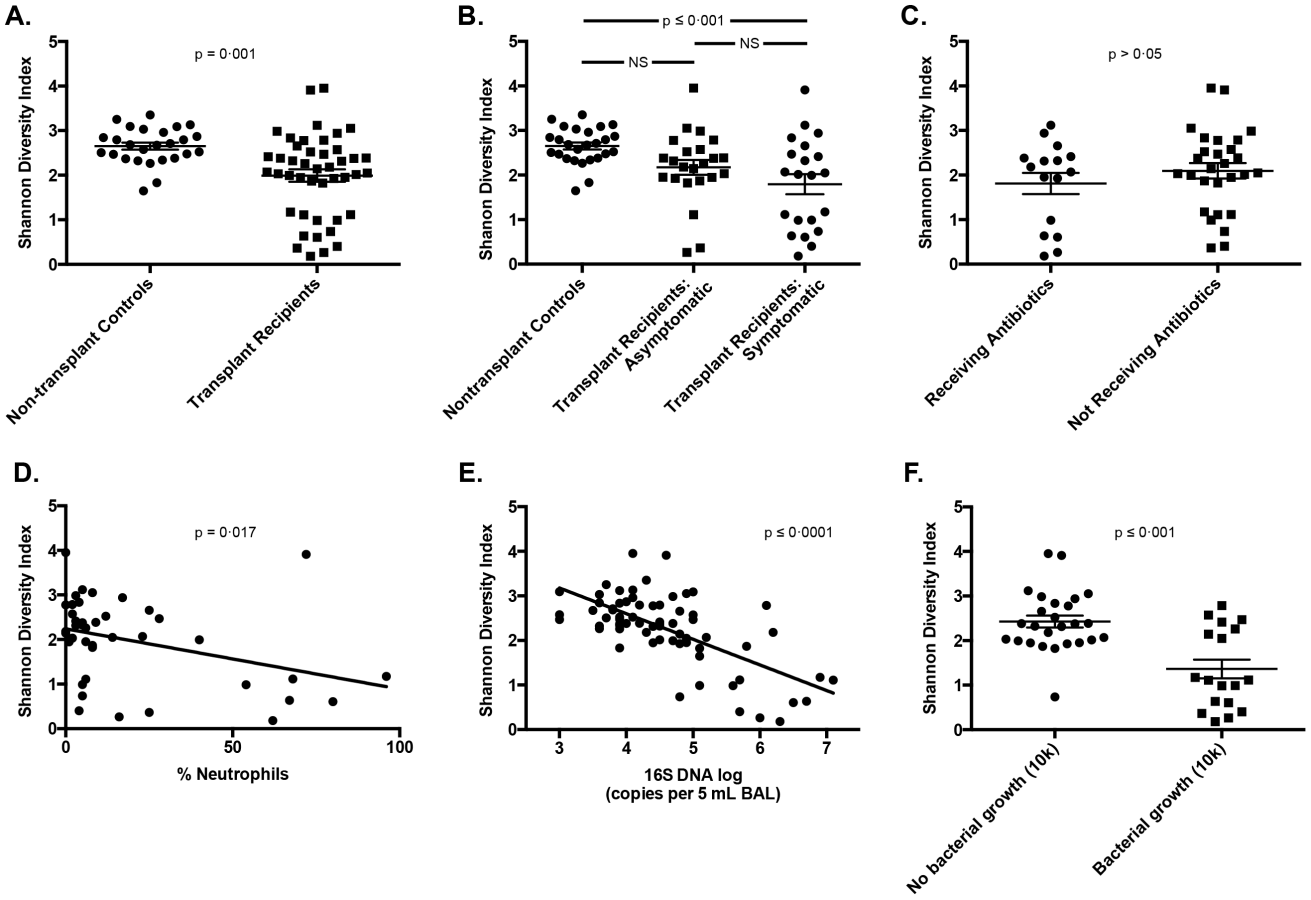
Shannon Diversity Indices of bacterial communities in BAL samples from lung transplant recipients. A-C: Comparison of bacterial community diversity by transplant status (A), specimen group (symptomatic/transplant status) (B) and antibiotic exposure (C). D-E: Bacterial community diversity correlated with BAL neutrophilia (D) and 16S DNA (E). F: Comparison of bacterial community diversity among transplant subjects by culture results. Comparison of group means performed with unpaired t-test and ANOVA with Tukey's multiple comparisons test. Continuous variables assessed for correlation with linear regression.

We also investigated microbial diversity in the BAL from transplant recipients. The overall microbial diversity, as measured by the Shannon diversity index, was decreased among lung transplant recipients as compared to non-transplant controls (p = 0.001, **Figure 4A**). The distribution of diversity among transplant subjects was not uniform on inspection of this figure, with most subjects comparable to nontransplant control subjects but with 12 (27.2%) exhibiting markedly decreased bacterial diversity. When specimens were analyzed according to bronchoscopy indication, diversity among non-transplant control subjects was significantly higher than that of symptomatic transplant recipients (p ≤ 0.001) but not that of asymptomatic transplant recipients (p>0.05) **(** **Figure 4B** **)**. Microbial diversity was not associated with exposure to antibiotics at the time of BAL or within seven days or 30 days of BAL (p>0.05 for all) (**Figure 4C**). Microbial diversity was significantly and negatively associated with the presence of BAL neutrophilia (p = 0.017) **(** **Figure 4D** **)**, bacterial DNA burden (p≤0.0001) **(** **Figure 4E** **)** and positive bacterial culture (p≤0.001) **(** **Figure 4F** **)**. Microbial diversity was not associated with any other tested clinical parameters, including pre-transplant diagnosis (**Table 4**). Thus, microbial diversity is not uniform in the lungs of transplant recipients and is negatively associated with other culture-independent indices of acute bacterial respiratory infection.

### BAL from Lung Transplant Recipients Contain Increased levels of Pseudomonadaceae, Enterobacteriaceae and Staphylococcaceae and Decreased Levels of Prevotellaceae, Veillonellaceae, and Streptococcaceae

Despite the statistically significant differences in the PCA clusters between subject groups (**Figure 3**), some overlap existed between 1) the non-transplant controls and asymptomatic transplant recipients as well as between 2) asymptomatic and symptomatic transplant recipients. To explore whether we could determine the microbial constituents that were responsible for clustering of specimens in the PCA, we utilized a biplot analysis of the PCA (**Figure 3D**). This revealed that one of the major factors accounting for the difference in the non-transplant control subjects and many asymptomatic transplant subjects compared to other subjects was the abundance of OTU 1054 (**Figure 3D** **, x**). This OTU was classified as a *Prevotella* sp.. The major factor accounting for the difference in the heterogenous symptomatic transplant recipient group was the abundance of a single OTU (1065) in five of the subjects. This OTU was classified as a *Pseudomonas* sp. (**Figure 3D** **, y**). Finally, the PCA region that included most asymptomatic and some symptomatic transplant recipients (**Figure 3D** **, z**) was largely defined by the presence of two other OTUs (1025 and 1080). OTU 1080 was classified as an *Escherichia* sp., while OTU 1025 was classified as a *Pseudomonas* sp., distinct from the other *Pseudomonas* OTU (1065). Thus, the bacteria identified in the collection of lung transplant recipient BALs in our study included at least two distinct *Pseudomonas* spp. that appeared to be differentially represented in asymptomatic vs. symptomatic subjects.

**Figure 5:**
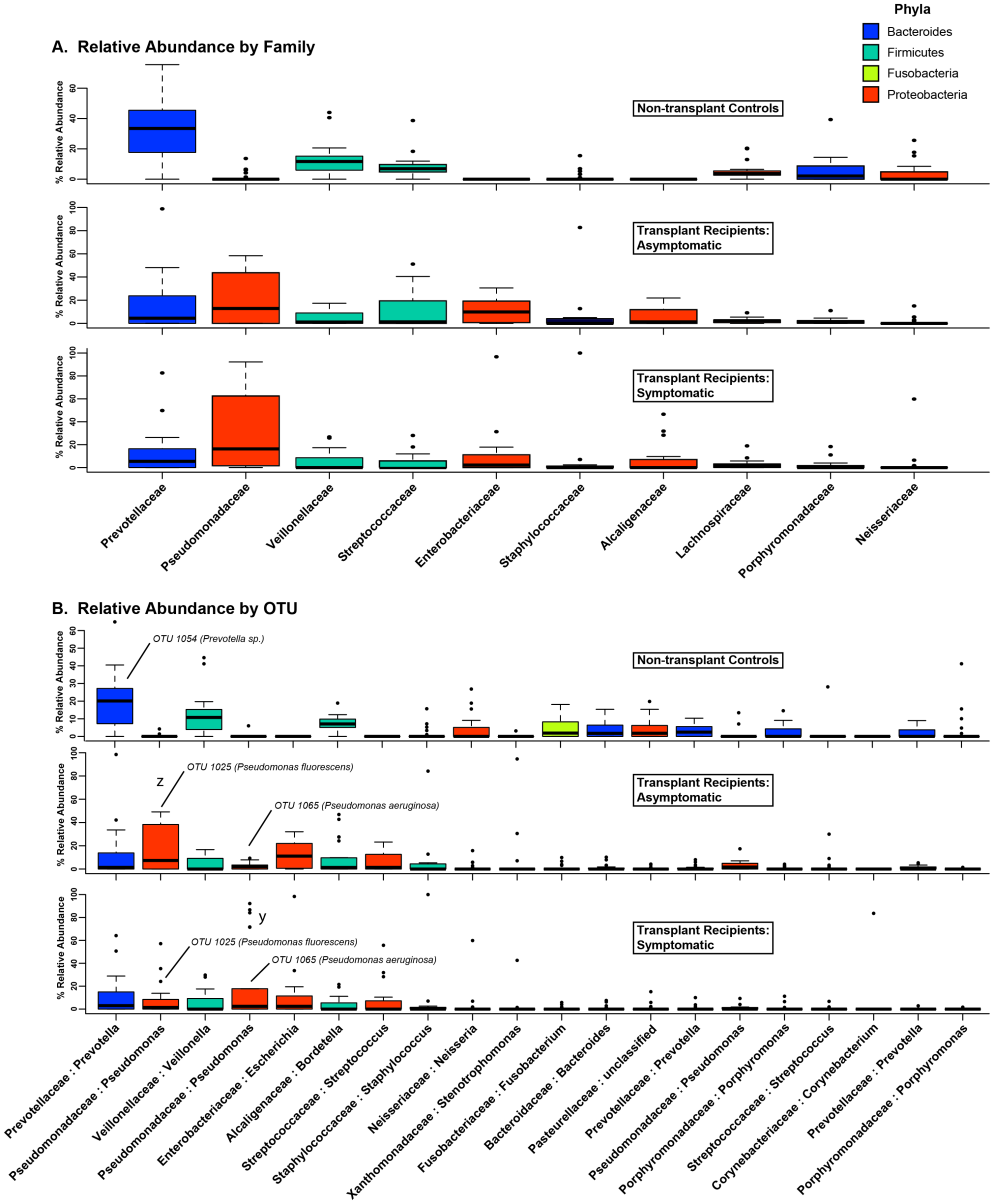
Taxonomic classification of the bacterial OTUs detected in BAL samples. Families and OTUs are ranked in descending order of mean relative abundance among all subjects. Box plots are colored according to phylum (see Phyla legend). Outliers are plotted as circles. A: Relative abundance of the 10 most abundant bacterial families. B: Relative abundance of 20 most abundant bacterial OTUs.

We next analyzed the differences in the BAL microbiota among our subject groups at a taxonomic level. At the levels of phylum and family taxonomic classification, symptomatic and asymptomatic transplant recipients were markedly distinct from non-transplant controls but indistinguishable from each other **(** **Figure 5A** **)**. Among non-transplant control subjects, the most commonly observed phyla (in descending order) were *Bacteroidetes*, *Firmicutes,* and *Proteobacteria*. These three phyla were also those most commonly observed among lung transplant recipients, though their relative frequency of detection was reversed, with *Proteobacteria* most commonly observed phylum. The most common bacterial families detected among non-transplant control subjects were *Prevotellaceae*, *Veillonellaceae,* and *Streptococcaceae*, while the most frequently detected families in BAL from transplant recipients were *Pseudomonadaceae, Enterobacteriaceae* and *Staphylococcaceae* **(** **Figure 5A** **)**. There was minimal difference at a family level of taxonomy between symptomatic and asymptomatic transplant recipients. The presence of *Pseudomonadaceae* in the lung transplant recipients, which was rare in control subjects**,** was reflected in the biplot PCA **(** **Figure 3D** **).** Overall, the majority of nontransplant control subjects had greater than 10% of their BAL microbiota consisting of *Prevotella* spp. and *Veillonella* spp., while this was infrequent among transplant subjects (**Table 5**). In contrast, the majority of transplant subjects had greater than 10% *Pseudomonas* spp. and *Escherichia* spp., which was infrequent among nontransplant control subjects (**Table 5**).

**Table 5:** Percentage of Subjects with >10% Relative Abundance of Prominent Genera.

Subject Group	*Prevotella*	*Veillonella*	*Streptococcus*	*Pseudomonas*	*Escherichia*
Nontransplant Control Subjects (26)	65.4%	53.8%	23.1%	3.8%	0.0%
Asymptomatic Transplant Subjects (23)	8.7%	17.4%	30.4%	52.2%	52.2%
Symptomatic Transplant Subjects (21)	4.8%	23.8%	14.3%	57.1%	28.6%

### Two Distinct Pseudomonas Species Can Be Identified in the BAL from Lung Transplant Recipients

The most abundant OTU among symptomatic transplant recipients was a *Pseudomonas* sp. (OTU 1065) that, when present, was found in very high relative abundance (**Table 6** **,** **Figure 5B** **, y; Figure S1**). This OTU was the same that defined the cluster of symptomatic transplant subjects in PCA analysis (**Figure 3B** **, y**). In contrast, the most commonly detected OTU among asymptomatic transplant recipients (OTU 1025) was separately classified also as a *Pseudomonas* sp. (**Table 6**, **Figure 5B** **, z**) and was the same OTU that was the major factor that accounted for the clustering of asymptomatic transplant subjects in the PCA analysis (**Figure 3B** **, z**). A third OTU (OTU 1024) was also classified as a *Pseudomonas* sp. but was relatively infrequent and was detected in low abundance compared to the other *Pseudomonas* OTUs (**Table 6** **, Figure S1**). A fourth *Pseudomonas*-classified OTU (OTU 955) was detected in only one specimen, for which it comprised only 0.19% of total reads; it was excluded from subsequent analysis. **Figure S2** shows the representative sequences for the three most abundant *Pseudomonas* OTUs as well as their phylogenetic relationships. As OTU 1024 was not a significant factor in the clustering of the samples by PCA, we focused our subsequent analysis on the two most common and significant *Pseudomonas* OTUs, 1025 and 1065.

**Table 6:** Percentage of Subjects with >10% Relative Abundance of Prominent Pseudomonas OTUs.

Subject Group	*OTU 1065: P. aeruginosa*	*OTU 1025: P. fluorescens*	*OTU 1024*
Nontransplant Control Subjects (26)	0.0%	0.0%	3.8%
Asymptomatic Transplant Subjects (23)	0.0%	47.8%	4.3%
Symptomatic Transplant Subjects (21)	28.6%	23.8%	0.0%

In order to more thoroughly characterize the *Pseudomonas* OTUs, we utilized the National Center for Biotechnology Information Basic Local Alignment Search Tool (BLAST) (http://blast.ncbi.nlm.nih.gov/Blast.cgi). We screened all fully sequenced microbial genomes within the NCBI database using the *Pseudomonas* OTUs' 97% homologous representative nucleotide sequences. Of the 10 speciated bacterial strains in the NCBI database sharing 100% coverage and homology with the representative sequence of the *P. aeruginosa* OTU (OTU 1065), nine were identified as *P. aeruginosa* (**Table 7**, **Table S1**). In contrast, the speciated bacterial strains sharing 100% coverage and homology with the other prominent *Pseudomonas* OTU (OTU 1025) were exclusively members of the *P. fluorescens* group (**Table 7**, **Table S1**, http://www.uniprot.org/taxonomy/200451).

**Table 7:** Abundance and Identification of Prominent Pseudomonas OTUs in Lung Transplant BALs**.**

OTU	% of BALs containing OTU	Mean abundance (SD)	Culture Results^1^	BLAST identification (100% coverage, 100% identity)
**1065**	66%	11.6% (25.6)	*P. aeruginosa*	*P. aeruginosa* ^2^
**1025**	61%	13.5% (18.0)	No growth	*P. fluorescens group* ^3^
**1024**	43%	1.9% (3.4)	No growth	None^4^

1: All BALs with OTU 1065 as the most abundant OTU grew *P. aeruginosa*. No BALs with either other OTU as the most abundant had positive cultures; 2: Of the 10 completed genomes in the database with 100% homology and 100% identity, nine were *P. aeruginosa*. 3: Of the two completed genomes in the database with 100% homology and 100% identity, one was *P. fluorescens* and one was *P. poae*, a recently described member of the *P. fluorescens* group (http://www.uniprot.org/taxonomy/200451); 4: No BLAST matches met 100% identity and homology. The genomes with the highest identity and homology were exclusively members of the *P. fluorescens* group.

Given the potential limitations of BLAST analysis in determining species-level taxonomic designations using 16S sequences, we designed a phylogenetic analysis utilizing existing sequences of clincally-obtained and reference genomes of 42 various *Pseudomonas* species as well as five non-*Pseudomonas* species. Using ten diferent housekeeping genes, we built MLST phylogeny trees (**Figure S3**) and compared them to trees generated using only 16S representative sequences (**Figure S2**). These trees illustrate that with these *Pseudomonas* species, MLST- and 16S-generated phylogenetic trees are similar. *P. aeruginosa* consistently separates from members of the *P. fluorescens* group in both, implying that enough genomic divergence is present in the 16S region to distinguish the two species. From our specimens, the *P. aeruginosa* OTU (OTU 1065) clustered exclusively with previously sequenced *P. aeruginosa* species. The other prominent *Pseudomonas* OTU (OTU 1025) clustered tightly and exclusively with members of the *P. fluorescens* group.

We also analyzed a number of the BAL samples with *P. aeruginosa* and *P. fluorescens*-specific PCR primers [26], [27]. Three BAL specimens from subjects with high abundance of OTU 1065 (and low OTU 1025) and five BAL specimens with high abundance of OTU 1025 (and low 1065) were analyzed. The specimens with high abundance of OTU 1065 were PCR-positive for *P. aeruginosa* while those with a high abundance of OTU 1025 were PCR-positive for *P. fluorescens*. All patients who had OTU 1065 as the singular dominant OTU in their BAL microbiota also grew *P. aeruginosa* from their BAL (using conventional clinical microbiology techniques). In contrast, *P. aeruginosa* was not grown from any of the 11 BALs for which OTU 1025 was the most abundant OTU. Thus, when considered in aggregate, the pyrosequencing, BLAST, phylogenetic-tree analysis, microbe-specific PCR and culture data all support the conclusion that OTU 1065 represents *P. aeruginosa* and OTU 1025 repressents *P. fluorescens*.

### The Presence of P. aeruginosa and P. fluorescens in the BAL of Lung Transplant Recipients are Associated with Distinct Clinical Features

**Figure 6:**
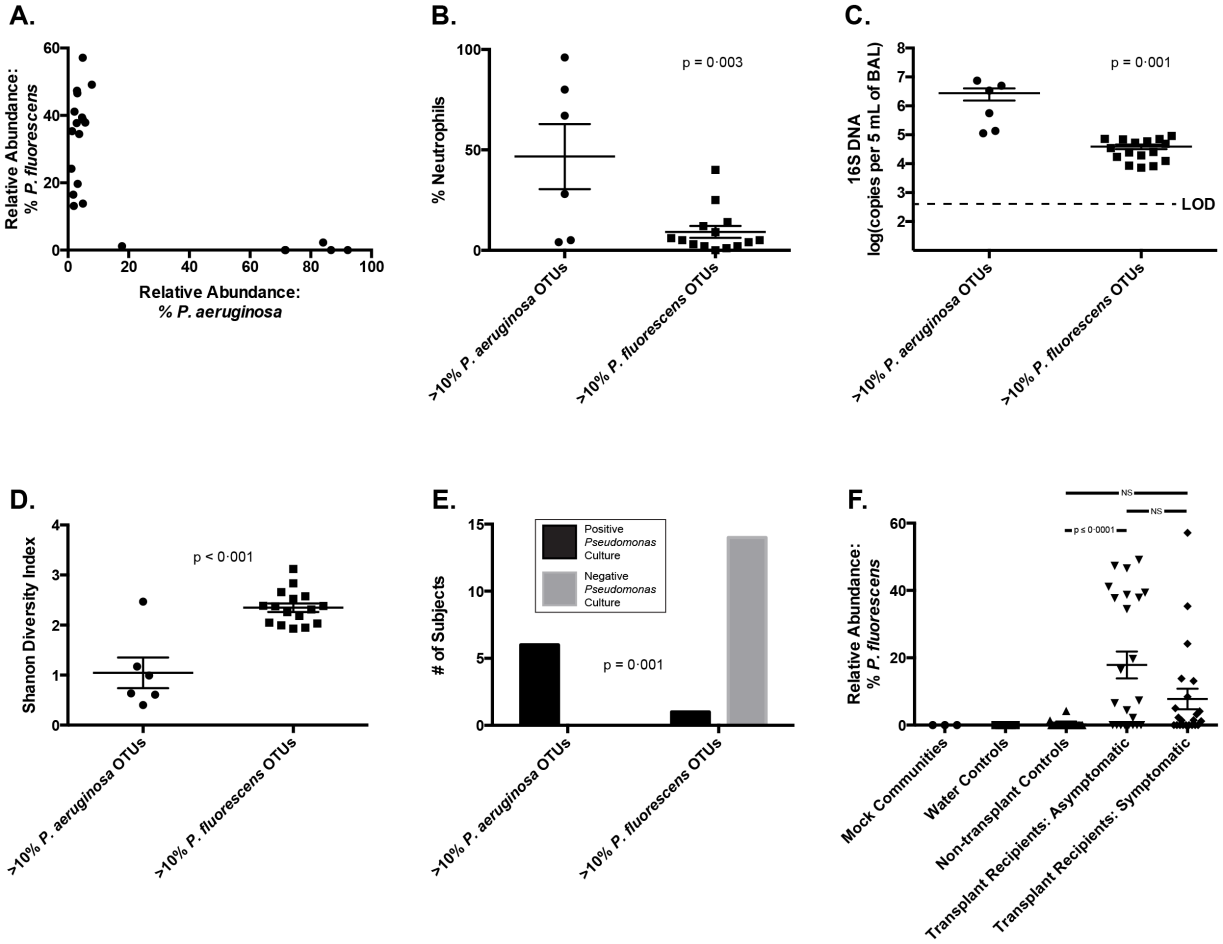
Clinical comparison among lung transplant recipients in relationship to two distinct *Pseudomonas* OTUs in BAL fluid. *P. aeruginosa* refers to OTU 1065, and *P. fluorescens* refers to OTU 1025 (see text). A: Relative abundance of each OTU in transplant recipients with greater than 10% of either. B-E: Comparison of *P. aeruginosa*-prominent and *P. fluorescens*-prominent BAL specimens by BAL neutrophilia (B), bacterial DNA burden (C), bacterial community diversity (D) and culture results (E). F: Relative abundance of *P. fluorescens* among pre-defined subject groups. Comparison of group means performed using unpaired t-test and ANOVA with Tukey's multiple comparisons test. Contingency testing performed using Fisher's exact test.

In order to assess potential differences in clinical significance associated with the presence of these two pseudomonads, we directly compared all subjects who had greater than 10% relative abundance of either prominent *Pseudomonas* OTU (**Figure 6**). No subjects had greater than 10% of both OTU (**Figure 6A**). When compared to subjects with greater than 10% *P. fluorescens*, subjects with *P. aeruginosa* had higher levels of BAL neutrophilia (**Figure 6B** **, p = 0.003**), bacterial DNA (**Figure 6C** **, p = 0.001**), and lower bacterial diversity (**Figure 6D** **, p<0.001**). All of the subjects with greater than 10% *P. aeruginosa* also had positive bacterial culture identified as *Pseudomonas*, while this was true of only one of the 15 subjects with greater than 10% *P. fluorescens* (**Figure 6E** **, p = 0.001**). Pre-transplant *Pseudomonas* infection was not more common among specimens with greater than 10% *P. aeruginosa* (2/6, 33%) than among other specimens (13/38, 34% (p>0.05). The most striking observation was that all of the subjects with greater than 10% *P. aeruginosa* were symptomatic at the time of bronchoscopy (**Table 6**), while most of the subjects with greater then 10% *P. fluorescens* were asymptomatic **(** **Figure 6F** **)**.

### Pseudomonas fluorescens has Strong Positive and Negative Correlations with Prominent Lung Microbes

**Figure 7:**
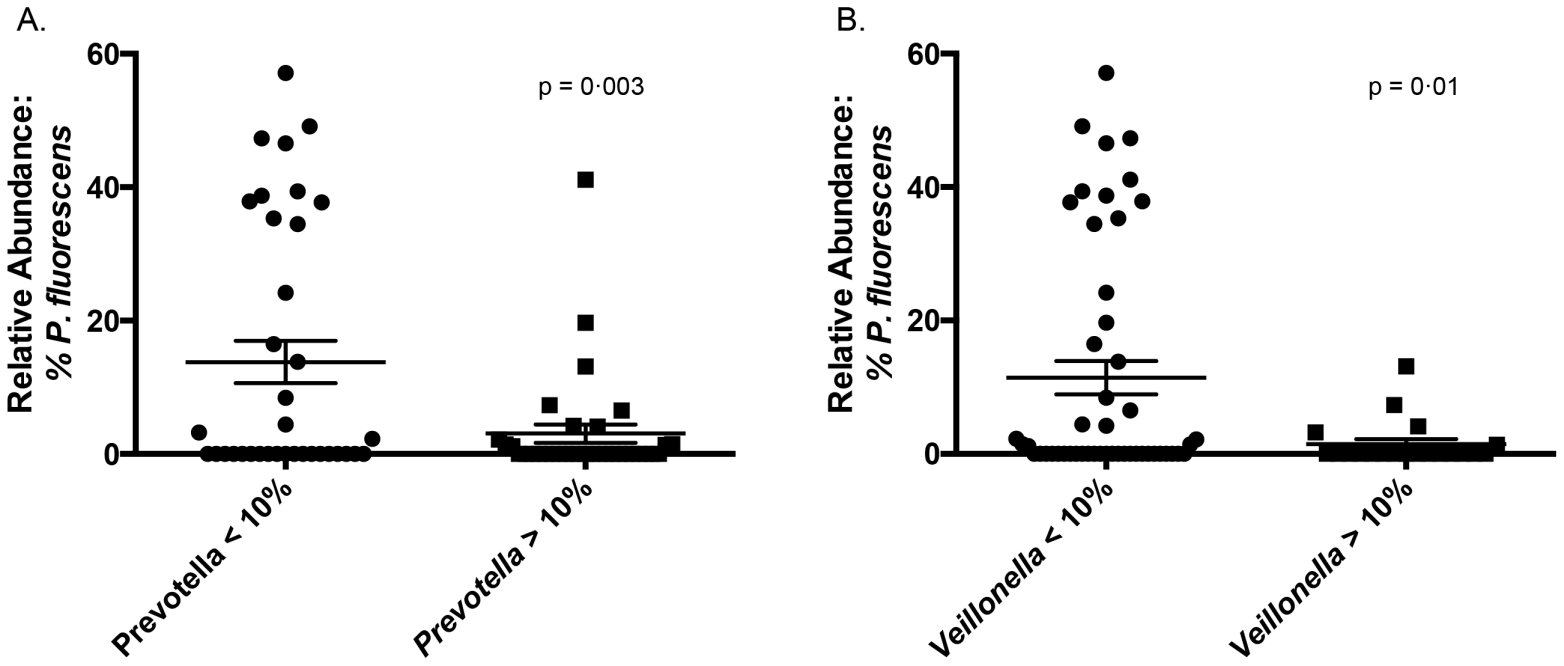
*In vivo* associations between *P. fluorescens* (OTU 1025) and other prominent bacteria: *Prevotella* (A) and *Veillonella* (B). Group means compared using unpaired t-test.

Given the known ability of *P. fluorescens* to produce numerous antimicrobial molecules, we investigated the presence of correlations between its relative abundance and that of other prominent lung microbes. Significant positive and linear associations were found between *P. fluorescens* and a common *Escherichia* OTU (OTU 1080) (p<0.0001, R^2^ = 0.37), and significant negative nonlinear associations were found between *P. fluorescens* and OTUs of *Prevotella* and *Veillonella* (p = 0.003 and 0.01, respectively) **(** **Figure 7** **)**. Among transplant subjects, *P. fluorescens* also had a significant, negative and nonlinear association with the presence of *P. aeruginosa* (p = 0.05, unpaired t test).

### Pseudomonas fluorescens is Commonly Isolated from the Airways of Lung Transplant Recipients and Patients with Other Chronic Lung Diseases

Given the prominence of *P. fluorescens* in the respiratory microbiota of our lung transplant recipients, we searched the database of bacterial culture isolates from the University of Michigan Clinical Microbiology Laboratory to determine how frequently it is cultured from respiratory specimens. Between January 1, 2002 and December 31, 2012, *P. fluorescens* was cultured from 242 distinct respiratory specimens, roughly 2 specimens per month. Of these, 59.9% were cultured using routine laboratory protocols for respiratory specimens; the remainder were cultured using a modified protocol adapted for specimens collected from patients with cystic fibrosis (CF). The majority (53.7%) were cultured from sputum specimens; 21.1% were cultured from throat swabs, and 13.2% were cultured from bronchoscopically-obtained specimens (BAL or brushings). The most common underlying condition was CF (38.8% of all isolates), followed by other chronic airway disease (COPD, asthma and non-CF bronchiectasis: 16.1%) and lung transplantation (7.4%, 18 specimens). 10.7% (26) of the specimens were obtained from patients with suspected acute pneumonia, and in all but four of these instances the patient was either chronically immunosuppressed or had recent healthcare exposures meeting criteria for healthcare-associated pneumonia. In most isolates (85.1%), *P. fluorescens* was co-isolated with species classified by the laboratory as "oral flora." Other species commonly co-isolated were *P. aeruginosa* (25.6%), *Staphylococcus aureus* (15.7%) and *Stenotrophomonas maltophilia* (11.6%). Thus despite the notable differences observed in the culturability of pseudomonads in our study, a global database search of UM Hospital Clinical Microbiology Laboratory records indicated that *P. fluorescens* is commonly isolated from respiratory specimens.

## Discussion

Using culture-independent techniques and BAL specimens from 59 total subjects, we identified that two distinct *Pseudomonas* species dominate the BAL microbiota of some transplant recipients. Whereas *P. aeruginosa*, when present, is detected in high abundance and is associated with clinical evidence of acute infection, *P. fluorescens* is commonly detected in moderate abundance and is rarely associated with parameters of acute infection. We observed that the decreased diversity of post-transplant lung microbiota is correlated with features of acute infection and is not uniform among transplant recipients.

Our results reveal that among post-transplant microbiota, at least two distinct *Pseudomonas* species are prominent and have widely divergent clinical associations, which provides a potential unifying explanation for the apparently dichotomous reports of the association between *Pseudomonas* and the development of BOS. Multiple independent studies have identified the presence of *P. aeruginosa* in respiratory cultures as a positive risk factor for the subsequent development of BOS [6], [12], [13]. Yet in the largest published study to date of lung transplant subjects utilizing culture-independent techniques of microbial identification, Willner et. al. observed a negative association between *Pseudomonas* spp. and the diagnosis of BOS [11]. While the subjects in our study with abundant *P. aeruginosa* exhibited evidence consistent with acute infection (symptoms of acute infection, BAL neutrophilia, increased bacterial burden, decreased bacterial diversity), the many subjects with abundant *P. fluorescens* exhibited little evidence of acute infection (**Figure 6B-6E**). The stark difference in culture positivity between these pseudomonads (**Figure 6E**) may explain the differences between prior culture-based studies and the culture-independent study by Willner et. al.. The distinctions between these two *Pseudomonas* species, while of clear clinical and biological significance, are unappreciated when analysis is limited to the family level of taxonomic designation (**Figure 5A**). Our results highlight the discriminatory power of incorporating additional techniques of microbial identification (e.g. culture, BLAST, phylogenetic tree generation, microbe-specific PCR) to complement the level of taxonomy provided by pyrosequencing and other culture-independent techniques.

The lack of detection of *P. fluorescens* via culture among our specimens was stark and surprising, especially given the relative frequency with which it is isolated in our clinical microbiology laboratory. Multiple explanations are possible. *P.fluorescens* is well-described to exist in a viable but not-culturable state in the environment^22,23^. All clinical respiratory specimens at our institution are incubated at 37**°**C, while the optimal growth temperature of P. fluorescens is below 32**°**C (a temperature range that is found in the airways [29]). *P. fluorescens* produces numerous antimicrobial metabolites and inhibits *in vitro* growth of other organisms^20,21^, and in a one report could only be cultured after the fluid containing it was dialyzed [30]. Thus its culture-negativity may reflect production of active culture inhibitors.

Our study is the first to describe the widespread abundance of *P. fluorescens* in the BAL of lung transplant recipients. We are aware of only one published report of the detection of *P. fluorescens* in BAL fluid (a single patient with ventilator-associated pneumonia, detected using 16S analysis and not via BAL culture) [31]. The *Pseudomonas* genus is large and contains an extremely diverse group of bacteria at the genomic level. Much work has been done on the phylogeny of this genus, and recent studies have demonstrated that *P. fluorescens* and *P. aeruginosa* characterize very distinct genomic groups [32]. *P. aeruginosa* strains, when analyzed using either multi-locus sequence typing (MLST) or 16S rRNA gene sequence comparisons, cluster tightly within a single related group, while analysis of *P. fluorescens* strains has identified three distinct genetic clades [33]. For this reason, *P. fluorescens* bacteria have been suggested to belong in a species-complex rather than a single species [34]. Members of this *P. fluorescens* species complex include all sequenced *P. fluorescens* genomes, along with numerous other speciated pseudomonads [33]. *P. poae*, encountered during our BLAST analysis (**Table S1**), is a fluorescent pseudomonad that is genetically included within the *P. fluorescens* group [32] and phylogenetic analysis within our laboratory using MLST of nine selected housekeeping genes also clusters it within the *P. fluorescens* species-complex (manuscript in preparation). Taken together, analysis using culture, 16S analysis, MLST and PCR all indicate that the two prominent *Pseudomonas* OTUs in lung transplant BAL represent two distinct pseudomonads: *P. aeruginosa* and *P. fluorescens*. Indeed, our review of *P. fluorescens* isolates from our own clinical microbiology laboratory found that the organism is cultured from respiratory specimens of patients with lung transplantation and other chronic lung diseases with relative frequency.

Other recent culture-independent studies have located *P. fluorescens* elsewhere in the aerodigestive tract. A recent culture-independent analysis of the human salivary microbiome found increased prevalence of *P. fluorescens* among solid organ transplant recipients, [35] and a separate study found *P. fluorescens* in the vast majority (93%) of gastric antrum biopsies taken from patients with acid-related gastrointestinal disorders [36]. Members of the *P. fluorescens* species-complex are obligate aerobic Gram-negative bacilli that are ubiquitous in plant, soil and water environments [37]; they are prominent plant commensals and are very rarely described as pathogenic in humans [38]. They have been commonly detected in the home environments of patients with respiratory disease [39]. Increased abundance of *P. fluorescens* has been observed in the oropharyngeal microbiota of patients infected with the H1N1 influenza virus [40]. Antibodies against *P. fluorescens* are common among patients with Crohn's disease [41], [42], though whether it is a marker or mediator of human disease remains undetermined.

The significant associations (both positive and negative) between abundance of *P. fluorescens* and that of other prominent bacteria has several possible interpretations. *P. fluorescens* actively inhibits in vitro growth of other bacteria [43]. It produces numerous antibiotics (including mupirocin [44]) and is used in agriculture as a biological control organism [37]. Hence, although the observed associations may be secondary to the presence of conditions that are jointly favorable to *P. fluorescens* and *Escherichia* sp. and unfavorable to *Prevotella* and *Veillonella* spp., an important research direction will be to determine whether *P. fluorescens* exhibits in vivo pro- and anti-biotic effects in the respiratory tract.

We observed decreased bacterial diversity in the BAL of transplant recipients when compared to controls and identified negative associations between BAL diversity and multiple clinical features associated with acute infection. Recent studies are divergent regarding changes in BAL microbiota diversity among lung transplant recipients when compared to non-transplant controls. Borewicz et al. observed increased BAL microbiota diversity among transplant recipients [15], while two other published reports reported decreased diversity [11], [14]. In COPD and asthma, the relationship between disease severity and lung microbial diversity has been similarly conflicting [1]. Most BAL microbiota studies to date have either excluded patients with active infection or have made no distinction in analysis between symptomatic and asymptomatic subjects (as in the above-cited lung transplant studies). Our findings are consistent with observations from the CF literature indicating that decreased diversity is not a direct consequence of duration of illness or impaired lung function but is instead driven by other factors [4].

To our knowledge, ours is the first report observing significant associations between BAL microbiota diversity and other BAL indices of acute infection. In our population, this decreased diversity did not appear to be secondary to recent exposure to antibiotics, as patients who had received antibiotics other than their *Pneumocystis* prophylaxis agent in the preceding 30 days, seven days and at the time of BAL did not have decreased BAL bacterial diversity indices. These results are surprising and divergent from the cystic fibrosis literature [4]. The fact that all transplant subjects are at baseline on antibiotics for *Pneumocystis* prophylaxis (unlike subjects with other lung pathologies such as CF) may be relevant to this difference. While decreased bacterial diversity in the gut has been associated with obesity and geography [45], [46], its significance in the lung apart from as a marker of acute infection is uncertain.

A potential limitation of this study is the absence of concurrent oral and nasal sampling for identification of "lung-specific" bacteria. There has been discussion in the literature about the extent to which microbiota detected in BAL reflect lung-resident organisms as opposed to oral-derived microbes picked up during sedation-related aspiration or broncho­scopic carryover. One study of six healthy subjects observed a similar constitution in the bacterial communities of the lower and upper respiratory tracts [20]; however, subsequent published studies from the NIH-funded LHMP consortium have identified bacterial species by BAL with significantly disproportionate representation in lower respiratory tract samples [16], [17]. These newer, larger studies suggest that some bacteria might replicate in or colonize the lower respiratory tract, with specific selective pressures favoring some species over others. Such preferential accumulation of certain OTUs supports the existence of lung-specific microbiota. An important contribution of the current study is the observation that route of bronchoscopy insertion (nasal or oral) has no appreciable effect on BAL microbiota (**Figure 2**) despite the markedly divergent microbial communities detected in the human mouth and nose [47]. This finding suggests that passage of the bronchoscope through these spaces has little impact on detected BAL microbiota, and bronchoscopic carryover of upper respiratory tract microbiota in BAL fluid is minimal. Further, the strong and significant associations we found between BAL bacterial community diversity and composition on one hand, and clinically and biologically significant parameters on the other, is relevant to ongoing discussions about the diagnostic implications of sampling lung microbiota via BAL [48]. We believe that the associations we report between BAL microbiota communities and significant clinical parameters indicate that the microorganisms detected in BAL specimens have clinical and biological significance, regardless of their derivation.

Another limitation of this study, common to all studies utilizing BAL, is the potentially variable effect of dilution on BAL assays. Despite efforts to standardize the procedure of saline instillation and collection, the BAL return in each bronchoscopy is variable. While this may result in variably concentrated samples, our results indicate that the variation in bacterial DNA burden among subjects is many-fold higher than that of variation in saline return (**Figure 1**), and any confounding effect of dilution is not enough to overwhelm the clear correlations apparent between bacterial DNA burden and important clinical parameters (**Figures 4E** **,** **6C**). Variation in dilution between BAL samples should have no impact on relative abundance of bacteria, from which most of this study's findings are derived. The use of 40 cycles in our PCR protocol is a potential source of bias, but our touchdown protocol (described in Methods) is optimized for low biomass samples and produces a low fraction of spurious priming [49].

Our results suggest that future analyses of the lung transplant microbiome should distinguish between *P. aeruginosa* and other *Pseudomonas* species, as well as between symptomatic and asymptomatic subjects and those with evidence of acute respiratory infection.

## Supporting Information

Figure S1 **Relative abundance of prominent** ***Pseudomonas*** **OTUs among lung transplant recipient BAL specimens.**
(TIF)

Figure S2 **Genomic Comparison of Prominent Pseudomonas OTUs.** A: Representative sequences of 16S V3-V5 for three prominent *Pseudomonas* OTUs. B: Phylogenetic tree derived from above sequences. Figures generated using Lasergene Megalign (Madison, MI).
(TIF)

Figure S3 **Phylogenetic Tree of** ***Pseudomonas*** **-classified OTUs and Clinically-Obtained and Reference Genomes.** MLST phylogeny tree created using DNASTAR SeqBuilder (Lasergene). Numbers in black are branch length. Numbers in blue are bootstrap confidence values.
(TIF)

Table S1 **NCBI BLAST Results for Prominent** ***Pseudomonas*** **OTUs.** Results restricted to sequences with 100% coverage and identity and ranked in descending order of total score.
(DOC)
