# Supplementary figures and images for: Changes in the Lung Microbiome following Lung Transplantation Include the Emergence of Two Distinct Pseudomonas Species with Distinct Clinical Associations
Source: PLoS One. 2014 May 15;9(5):e97214. doi: 10.1371/journal.pone.0097214 (PMC4022512; doi:10.1371/journal.pone.0097214)

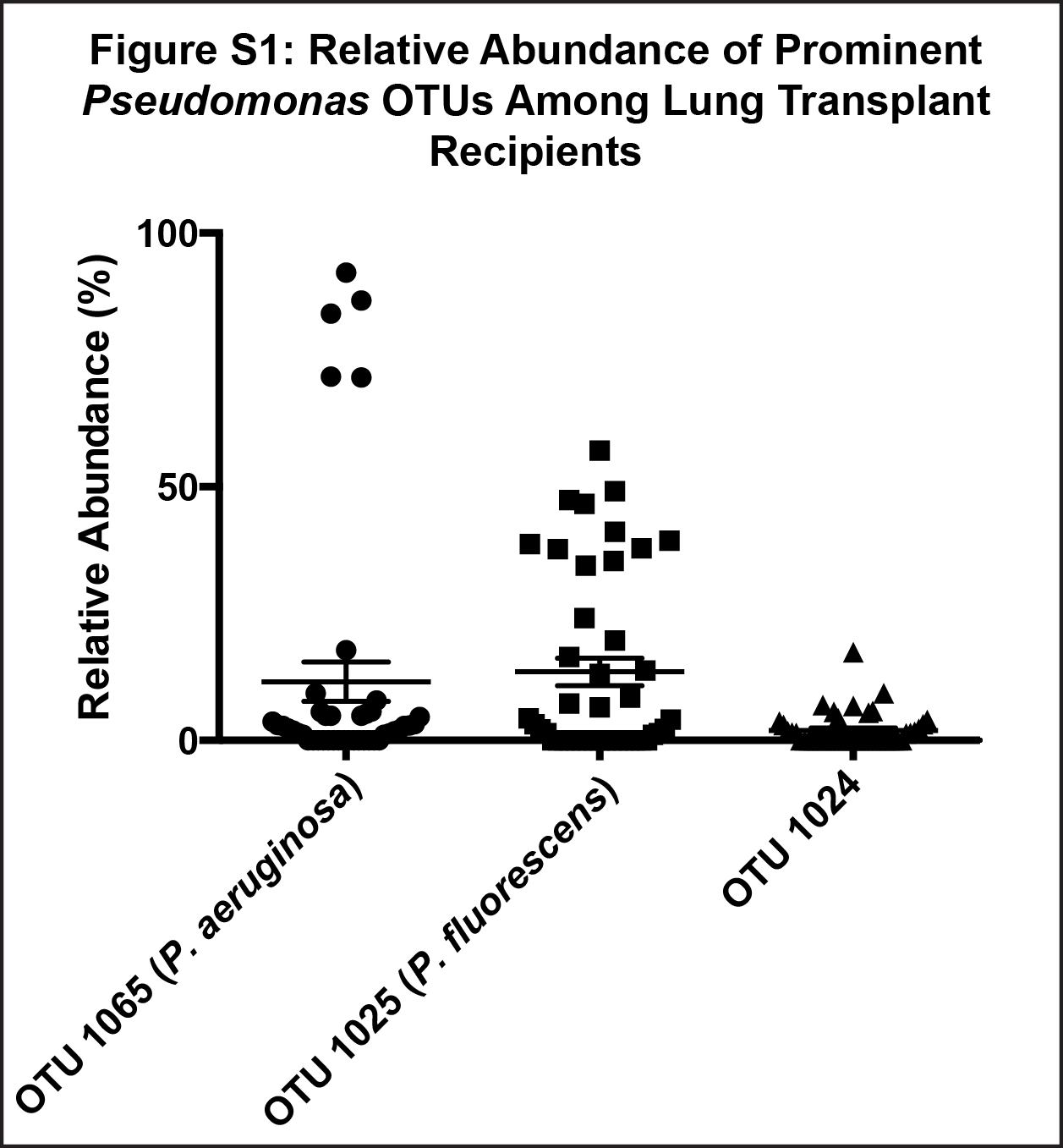

Supplement: Figure S1 — Relative abundance of prominent Pseudomonas OTUs among lung transplant recipient BAL specimens. (TIF) [file pone.0097214.s001.tif]

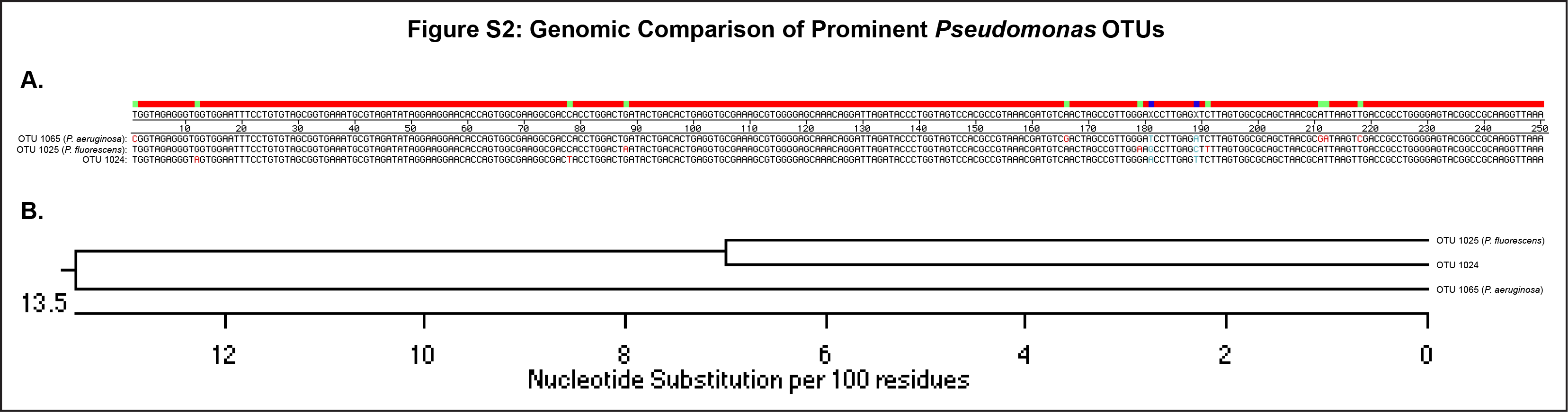

Supplement: Figure S2 — Genomic Comparison of Prominent Pseudomonas OTUs. A: Representative sequences of 16S V3-V5 for three prominent Pseudomonas OTUs. B: Phylogenetic tree derived from above sequences. Figures generated using Lasergene Megalign (Madison, MI). (TIF) [file pone.0097214.s002.tif]

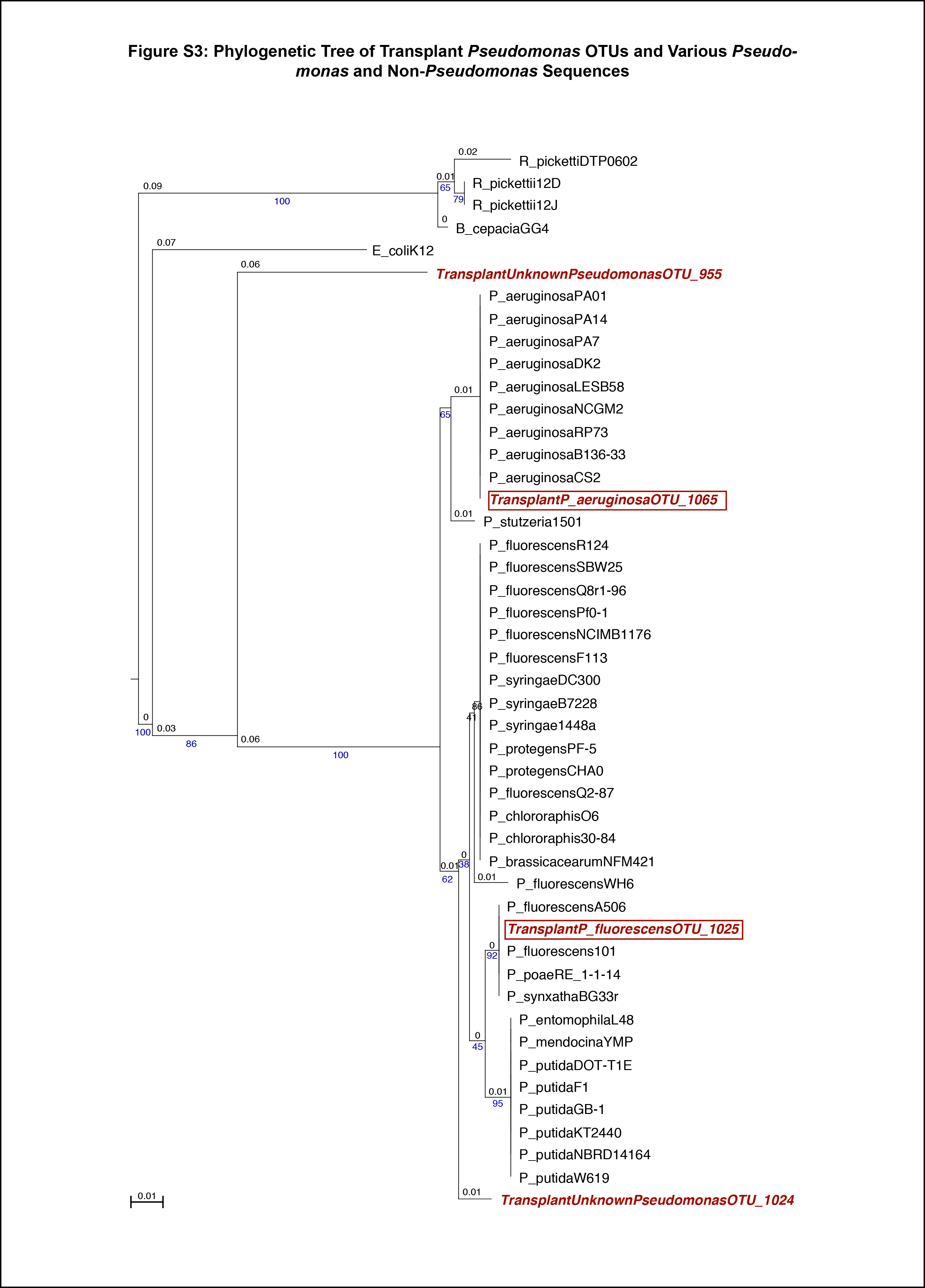

Supplement: Figure S3 — Phylogenetic Tree of Pseudomonas -classified OTUs and Clinically-Obtained and Reference Genomes. MLST phylogeny tree created using DNASTAR SeqBuilder (Lasergene). Numbers in black are branch length. Numbers in blue are bootstrap confidence values. (TIF) [file pone.0097214.s003.tif]
