# Supplementary material for: Changes in the Lung Microbiome following Lung Transplantation Include the Emergence of Two Distinct Pseudomonas Species with Distinct Clinical Associations
Source: PLoS One. 2014 May 15;9(5):e97214. doi: 10.1371/journal.pone.0097214 (PMC4022512; doi:10.1371/journal.pone.0097214)
Supplement: Table S1 — NCBI BLAST Results for Prominent Pseudomonas OTUs. Results restricted to sequences with 100% coverage and identity and ranked in descending order of total score. (DOC) [file pone.0097214.s004.doc]

| **Table S1: NCBI BLAST Results for Prominent *Pseudomonas* OTUs** | | | | | | | |
| --- | --- | --- | --- | --- | --- | --- | --- |
|  | **Description** | **Max score** | **Total score** | **Query cover** | **E value** | **Identity** | **Accession** |
| **OTU 1065** | *Pseudomonas denitrificans* ATCC 13867 | 462 | 2313 | 100% | 1E-127 | 100% | NC_020829.1 |
|  | *Pseudomonas aeruginosa* RP73 | 462 | 1851 | 100% | 1E-127 | 100% | NC_021577.1 |
|  | *Pseudomonas aeruginosa* B136-33 | 462 | 1851 | 100% | 1E-127 | 100% | NC_020912.1 |
|  | *Pseudomonas aeruginosa* DK2 chromosome | 462 | 1851 | 100% | 1E-127 | 100% | NC_018080.1 |
|  | *Pseudomonas aeruginosa* NCGM2.S1 chromosome 1 | 462 | 1851 | 100% | 1E-127 | 100% | NC_017549.1 |
|  | *Pseudomonas aeruginosa* M18 chromosome | 462 | 1851 | 100% | 1E-127 | 100% | NC_017548.1 |
|  | *Pseudomonas aeruginosa* LESB58 chromosome | 462 | 1851 | 100% | 1E-127 | 100% | NC_011770.1 |
|  | *Pseudomonas aeruginosa* UCBPP-PA14 chromosome | 462 | 1851 | 100% | 1E-127 | 100% | NC_008463.1 |
|  | *Pseudomonas aeruginosa* PA7 chromosome | 462 | 1840 | 100% | 1E-127 | 100% | NC_009656.1 |
|  | *Pseudomonas aeruginosa* PAO1 chromosome | 462 | 1840 | 100% | 1E-127 | 100% | NC_002516.2 |
| **OTU 1025** | *Pseudomonas fluorescens* A506 chromosome | 462 | 2776 | 100% | 1E-127 | 100% | NC_017911.1 |
|  | *Pseudomonas poae* RE*1-1-14 | 462 | 2313 | 100% | 1E-127 | 100% | NC_020209.1 |
